# Microwave-Assisted Extraction of Oxymatrine from *Sophora flavescens*

**DOI:** 10.3390/molecules16097391

**Published:** 2011-08-30

**Authors:** En-Qin Xia, Bo Cui, Xiang-Rong Xu, Yang Song, Xu-Xia Ai, Hua-Bin Li

**Affiliations:** 1 Guangdong Provincial Key Laboratory of Food, Nutrition and Health, School of Public Health, Sun Yat-Sen University, Guangzhou 510080, China; Email: enqinxia@163.com (E.-Q.X.); cuibno@163.com (B.C.); sssongyang@163.com (Y.S.); 20040995axx@163.com (X.-X.A.); 2 Key Laboratory of Marine Bio-resources Sustainable Utilization, South China Sea Institute of Oceanology, Chinese Academy of Sciences, Guangzhou 510301, China; Email: xuxr2000@yahoo.com (X.-R.X.)

**Keywords:** microwave, extraction, oxymatrine, *Sophora flavescens*

## Abstract

In this paper, microwave-assisted extraction (MAE) of oxymatrine from *Sophora flavescens* were studied by HPLC-photodiode array detection. Effects of several experimental parameters, such as concentration of extraction solvent, ratio of liquid to material, microwave power, extraction temperature, and extraction time on the extraction efficiencies of oxymatrine were evaluated. The optimal extraction conditions were 60% ethanol, a 20:1 (v/v) ratio of liquid to material and extraction for 10 min at 50 °C under 500 W microwave irradiation. Under the optimum conditions, the yield of oxymatrine was 14.37 mg/g. The crude extract obtained could be used as either a component of some complex traditional medicines or for further isolation and purification of bioactive compounds. The results, which indicated that MAE is a very useful tool for the extraction of important phytochemicals from plant materials, should prove helpful for the full utilization of *Sophora flavescens*.

## 1. Introduction

*Sophora flavescens* Ait has been widely used for the treatment of cancer, hepatitis, leucopenia, bronchitis, cardiac and skin diseases [1,2,3,4,5]. The main bioactive component of *Sophora flavescens* is oxymatrine, which possesses important biological activities, such as anticancer and inhibition of hepatitis B virus replication [5,6,7]. Oxymatrine can be extracted from this plant by several methods, such as maceration extraction, the decocting, refluxing or leakage methods and supercritical fluid extraction [8,9,10,11,12]. Although methanol-water and chloroform-ammonia solutions were also used as extracting solvents for extraction of oxymatrine from *Sophora flavescens* in the literature [8,13], water and ethanol-water solutions were more widely used because of the toxicity of chloroform and methanol, which are harmful to human beings and environment. The extraction temperature range used in the literature was from room temperature (about 25 °C) to 100 °C [8,9,10,11,12]. However, the extraction rates of oxymatrine were usually low using conventional methods, such as the decocting method (52.3%) and refluxing method (53.4%) [8]. On the other hand, microwave-assisted extraction (MAE) has been successfully developed in recent years for the extraction of some phytochemicals from plant materials [14,15,16,17,18,19,20,21,22,23,24], and the extraction rate could be improved greatly [25]. Microwave-assisted extraction heats the extraction solvent quickly, and accelerates the extraction process for desorption of the targeted compounds from the matrix. Therefore, MAE is a highly efficient and reduced solvent- and time-consumption method. However, it was unknown whether the extraction rate of oxymatrine could be improved using MAE. In this paper, the effects of microwave irradiation on the extraction of oxymatrine from *Sophora flavescens* were studied. The results obtained should be helpful for the full utilization of *Sophora flavescens*.

## 2. Results and Discussion

### 2.1. Effect of Ethanol Concentration on the Extraction of Oxymatrine

**Figure 1:**
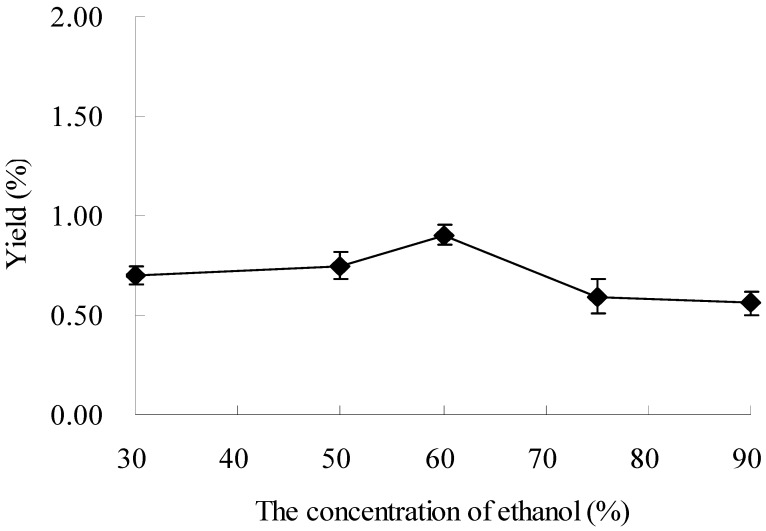
Effect of ethanol concentration on the extraction yield.

In the present study, ethanol-water solutions at different ratios were tested as extraction solvent for extraction of oxymatrine from *Sophora flavescens* under microwave irradiation. Other experimental parameters were set as follows: the ratio of liquid to material, 10:1 (mL/g); microwave power, 500 W; extracting temperature, 25 °C; and extracting time, 20 min. The results revealed that the extraction yield was the highest when 60% ethanol was used as extracting solvent (Figure 1). Seen from Figure 1, the yields increased when the concentration of ethanol increased from 50% to 60% (*p* < 0.1). Then, the yields declined from 60% to 90% ethanol. These results supported previous findings that the concentration of organic solvent played an important role in the extraction of bioactive components from plant materials [26,27]. The results indicated that 60% ethanol was suitable for the microwave-assisted extraction of oxymatrine from the plant, which was chosen as extracting solvent in the further experiments.

### 2.2. Effect of the Ratio of Liquid to Material on the Extraction of Oxymatrine

**Figure 2:**
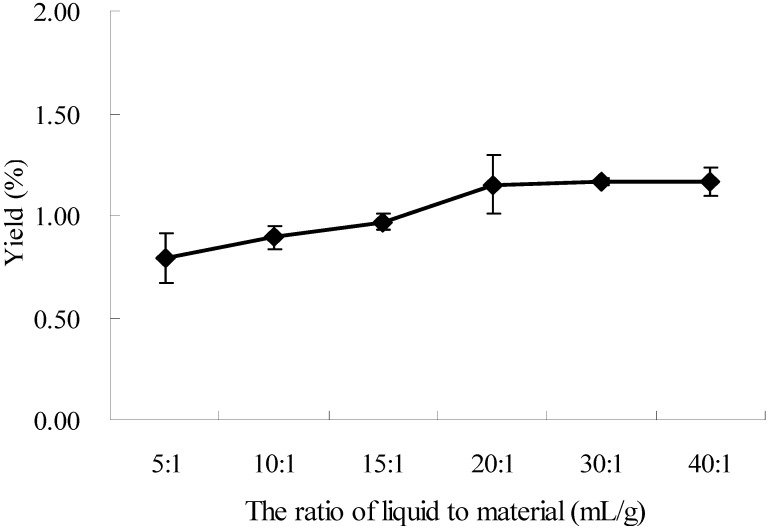
Effect of the ratio of liquid to material on the extraction yield.

In order to obtain the maximum extraction yield, effects of the ratio of liquid to material on the extracted yield of oxymatrine were studied. The amount of solvent was changed to examine different solvent/mass ratios. Other experimental parameters were 60% ethanol, extracting temperature at 25 °C, and 20 min of microwave irradiation with 500 W. The results were displayed in Figure 2. The extraction yield increased with the increased ratio of liquid to material from 5:1 to 20:1, and remained almost unchanged from 20:1 to 40:1. The extraction yield increased about 50% when the ratio of liquid to material increased from 5:1 to 20:1, which indicated that the ratio of liquid to material has a significant effect on the extraction efficiency of oxymatrine (*p* < 0.1). The ratio of 20:1 was chosen as the optimal ratio of liquid to material.

### 2.3. Effect of Microwave Power on the Extraction of Oxymatrine

**Figure 3:**
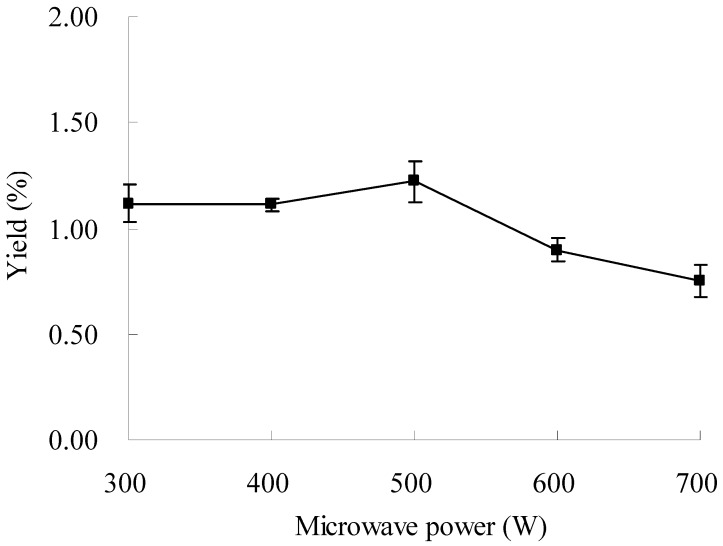
Effect of microwave power on the extraction yield.

Effects of microwave power on the yield of oxymatrine were investigated under the conditions of 60% ethanol, the ratio of liquid to material at 20:1, the extraction time of 20 min, and the extraction temperature at 25 °C. The results are shown in Figure 3. The yield of oxymatrine remained almost unchanged from 300 to 400 W, and then increased slightly from 400 to 500 W. The yield of oxymatrine decreased with increasing microwave power from 500 to 700 W. The highest yield was obtained at 500 W (Figure 3). The microwave can accelerate the extraction process for desorption of the targeted compounds from matrix at low power, and may induce decomposition of some target molecules at high power [28]. Therefore, 500 W of microwave power was used in the further experiments.

### 2.4. Effect of Temperature on the Extraction of Oxymatrine

**Figure 4:**
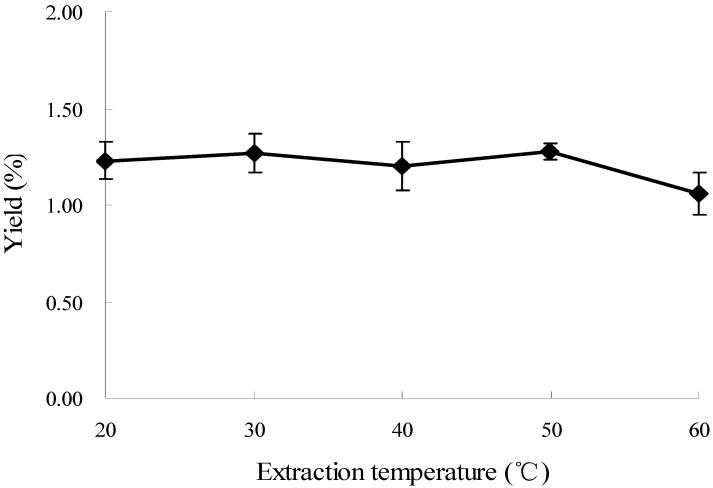
Effect of temperature on the extraction yield.

Effects of extraction temperature on the yield of oxymatrine were evaluated at different temperatures (20, 30, 40, 50, 60 °C), and other experimental parameters were 60% ethanol, the ratio of liquid to material at 20:1, and the extraction time of 20 min with a microwave power of 500 W. The results are shown in Figure 4. The effects of extraction temperature on the yield of oxymatrine were slight in the temperature range tested, and 50 °C was used in the subsequent experiments.

### 2.5. Effect of Time on the Extraction of Oxymatrine

**Figure 5:**
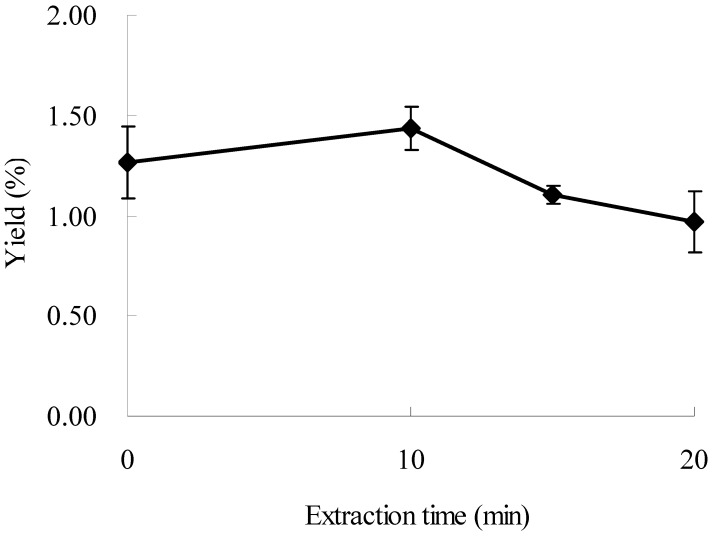
Effect of time on the extraction yield.

Effect of extraction time on the yield of oxymatrine was tested, and other experimental parameters were 60% ethanol with a ratio of liquid to material of 20:1, and extraction temperature at 50 °C under microwave irradiation of 500 W. The results are displayed in Figure 5. The yield of oxymatrine increased from 0 to 10 min (*p* < 0.1), and then decreased with the increase of irradiation time after 10 min. The maximum yield was obtained at 10 min, which was 14.37 ± 0.48 mg/g. The results indicated that microwave might accelerate the extraction of oxymatrine from plant material in a short time (10 min), and oxymatrine could be degraded after a longer time, which led the yield to decrease.

Effects of several parameters on extraction yield of oxymatrine were summarized in Table 1. Seen from Table 1, effects of ratio of liquid to material (L/M) and time on extraction yield of oxymatrine were bigger than ethanol concentration, microwave power (MW power) and temperature (Temp).

**Table 1:** Effects of experimental parameters on extraction yield of oxymatrine.

Factor	Optimum	Ethanol (%)	L/M	MW power (w)	Temp (°C)	Time (min)	Yield (mg/g)
Ethanol	60	-	10:1	500	25	20	9.03 ± 0.39
L/M	20:1	60	-	500	25	20	11.52 ± 0.98
MW power	500	60	20:1	-	25	20	12.19 ± 0.52
Temp	50	60	20:1	500	-	20	12.81 ± 0.22
Time	10	60	20:1	500	50	-	14.37 ± 0.48

### 2.6. Comparison of Microwave-Assisted Extraction with Conventional Extraction Methods

For comparison, Soxhlet extraction was carried out at 90 °C for 4 h and conventional solvent extraction was conducted at 25 °C for 24 h. If the extraction rate of oxymatrine obtained by MAE at 50 °C for 50 min was defined as 100% (Table 2), the rates obtained by conventional solvent extraction and Soxhlet extraction were 77.6% and 90.7%, respectively.

**Table 2:** Effects of extraction times on extraction rate of oxymatrine.

Extraction times	Microwave-assisted extraction (10 min)		Soaking 30 min without MAE	
	Extraction rate (%)	RSD (%) (n = 3)	Extraction rate (%)	RSD (%) (n = 3)
1	**65.41**	**3.71**	**58.62**	**4.9**
2	30.78	0.80	8.74	1.9
3	2.57	1.61	4.20	7.0
4	0.93	8.82	0.97	9.8
5	0.32	9.65	0.38	8.6
Total	100		72.91	

That is, the extraction rate of oxymatrine by MAE was higher than those by conventional solvent extraction and Soxhlet extraction. Furthermore, the extraction rate of oxymatrine obtained by MAE was also higher than those (52.3%–53.4%) obtained using decocting method and refluxing method in the literature [8], although MAE was carried out under the conditions of lower temperature and a shorter time. Compared with soaking 30 min without MAE (Table 2), effect of MAE on the recovery was obvious if extraction times were 2 or 3 (*p* < 0.1). In addition, no selectivity of extraction was observed by comparison of chromatograms with or without microwave irradiation.

## 3. Experimental

### 3.1. Chemicals

Oxymatrine (98% purity) was bought from Siyi Biotechnology Company (Chengdu, China). Ethanol, methanol and acetonitrile were HPLC grade and purchased from Merck (Darmstadt, German). Phosphoric acid was bought from Tianjin Chemical Reagent Company (Tianjin, China). Deionized water was used throughout the experiment. A stock solution of oxymatrine (10 mg/mL) was prepared in methanol and was stored at 4 °C. The calibration standards (10–500 μg/mL) were prepared from the stock solution by the serial dilution of methanol.

### 3.2. Instruments

The microwave-assisted extraction was carried out in an X-100A microwave extraction device (Xianghu Instrumental Company, Beijing, China) with a microwave power of 1,000 W and equipped with a temperature monitor as well as microprocessor programmer software to control the performance parameters of the microwave device, *i.e.*, microwave power, temperature and running time.

### 3.3. Plant Material and Sample Treatment

Dried roots (with humidity of 1.8%) of *Sophora flavescens* Ait were purchased from a drugstore in Guangzhou (Guangdong Province, China), ground into powder in a knife mill, and then stored at 4 °C in a refrigerator. The root powder (1 g) was accurately weighed, placed in a capped glass tube, and then mixed with an appropriate amount of extraction solvent. After soaking for 30 min that permitted solvent to wet the plant material, the tube with the sample was immersed into the water bath of the microwave device, and irradiated under the pre-set microwave power, extraction temperature and time conditions. After extraction, the sample was centrifuged at 9,600 g for 10 min, and then the supernatant was collected for HPLC analysis. For comparison, Soxhlet extraction was carried out with a Soxhlet extraction apparatus at 90 °C for 4 h, and conventional solvent extraction was conducted with a mechanic shaker at room temperature (25 °C) for 24 h. The extraction solvent was 60% ethanol, and the ratio of liquid to material was 20:1.

### 3.4. HPLC Analysis

**Figure 6:**
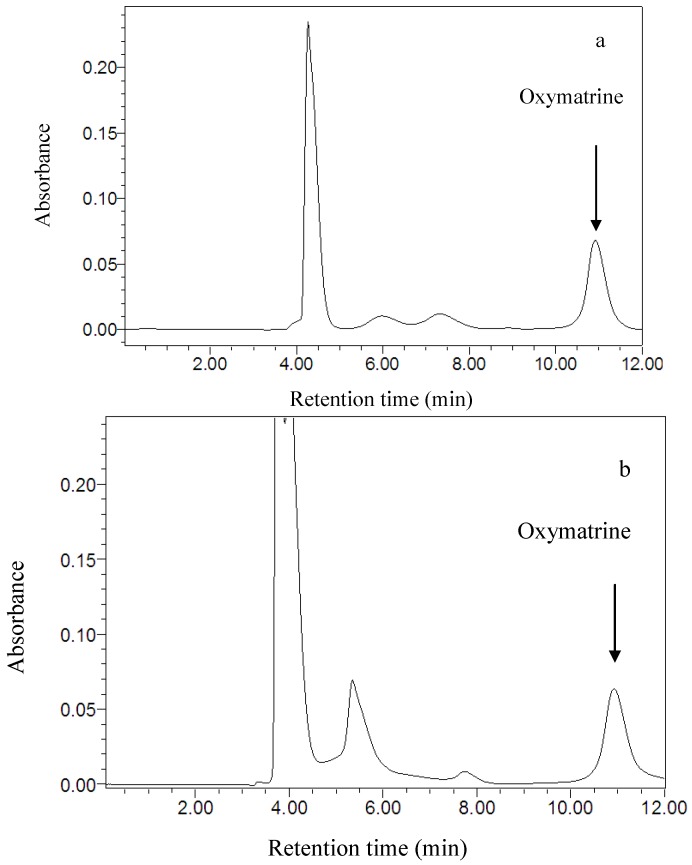
Chromatograms of oxymatrine in standard solution (**a**) and in the sample (**b**).

A Waters (Milford, MA, USA) 1525 binary HPLC pump separation module equipped with a Waters 2,996 photodiode array detector was used. An Agilent Zorbax Extend-C18 column (250 mm × 4.6 mm, 5 μm) and an auto-injector (10 μL) were also used. The mobile phase was acetonitrile and 0.05 mol/L solution of phosphoric acid in water (10:90, v/v) with a flow-rate of 1 mL/min [4]. The UV spectra were recorded between 190 and 400 nm for peak characterization, and the detection wavelength was set at 210 nm. The column temperature was kept at 27 °C. The peak area was used to calculate the amount of oxymatrine from the standard curve. The chromatograms of oxymatrine in standard solution and in the sample are shown in Figure 6.

All the experiments were conducted in triplicate, and the average values ± SD (standard deviation) were reported.

## 4. Conclusions

A microwave-assisted extraction method has been developed for the extraction of oxymatrine from *Sophora flavescens*. Microwave irradiation was a powerful tool, which efficiently improved the extraction of oxymatrine. Effects of several experimental parameters on the extracting yields of oxymatrine have been evaluated, and the optimal extraction conditions were determined as 60% ethanol, a ratio of liquid to material at 20:1, and the extraction for 10 min at 50 °C under 500 W microwave irradiation. Under the optimal conditions, the yield of oxymatrine was 14.37 mg/g. The crude extract could be used as either components of some complex traditional medicines or for further isolation and purification of oxymatrine. The results obtained are helpful for the full utilization of *Sophora flavescens*, which also indicated that microwave-assisted extraction is a very useful tool for the extraction of important bioactive compounds from plant materials.
